# Successful cholangioscopy-guided cannulation using a novel slim cholangioscope in a patient with Roux-en-Y gastrectomy

**DOI:** 10.1055/a-2244-4160

**Published:** 2024-02-15

**Authors:** Yuki Tanisaka, Masafumi Mizuide, Akashi Fujita, Takahiro Shin, Kei Sugimoto, Ryuhei Jinushi, Shomei Ryozawa

**Affiliations:** 1 183786 Gastroenterology, Saitama Medical University International Medical Center, Hidaka, Japan

Selective biliary cannulation in patients with Roux-en-Y gastrectomy is technically challenging 1 2 . Peroral cholangioscopy (POCS) is beneficial for direct visualization of intraductal bile duct lesions 3 . Recent studies have confirmed the viability of cholangioscopy-guided cannulation 4 . We report a successful cholangioscopy-guided cannulation achieved using a novel slim cholangioscope in a patient with Roux-en-Y gastrectomy.

**Fig. 1:**
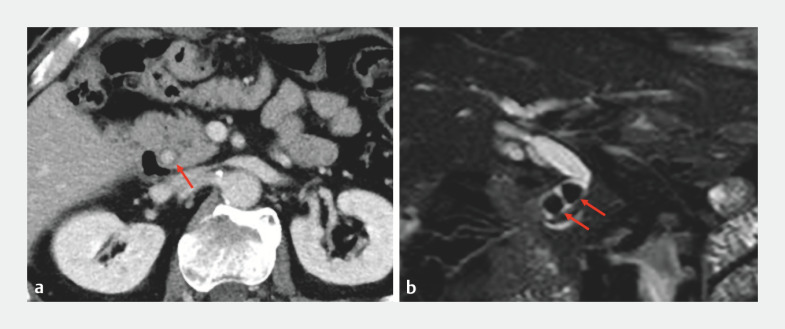
Images showing stones in the common bile duct (red arrows) on: **a** computed tomography; **b** magnetic resonance imaging.

**Fig. 2:**
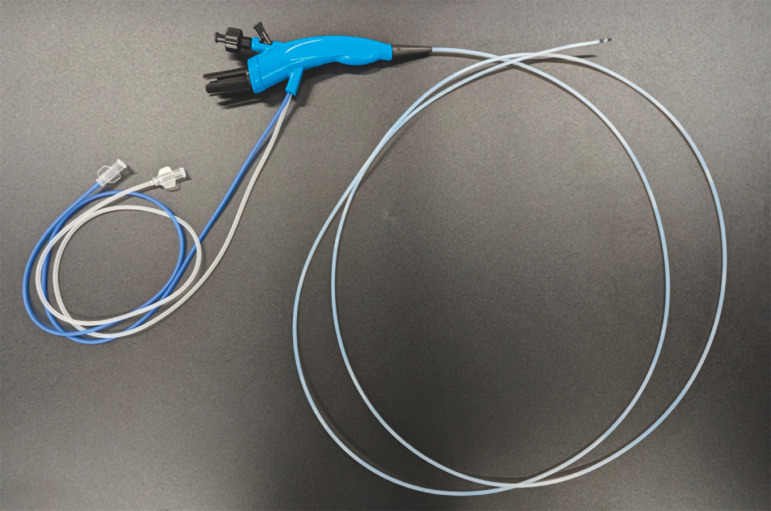
Photograph of the slim pancreatoscope (DRES Slim Scope; Japan Lifeline, Japan), which measures 195 cm in length, with scope and tip diameters of 2.6 mm and 2.3 mm, respectively.

**Fig. 3:**
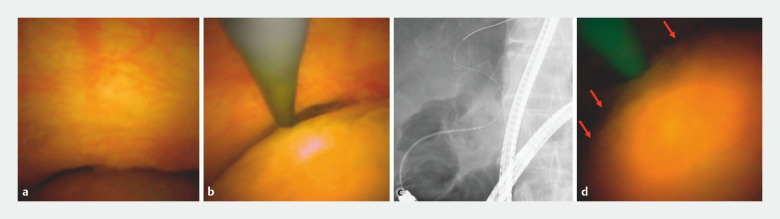
Cholangioscopic and cholangiographic images showing: **a,b** the bile duct mucosa, prompting the advancement of a 0.025-inch guidewire; **c** successful biliary cannulation; **d** a stone in the common bile duct (red arrows).

**Fig. 4:**
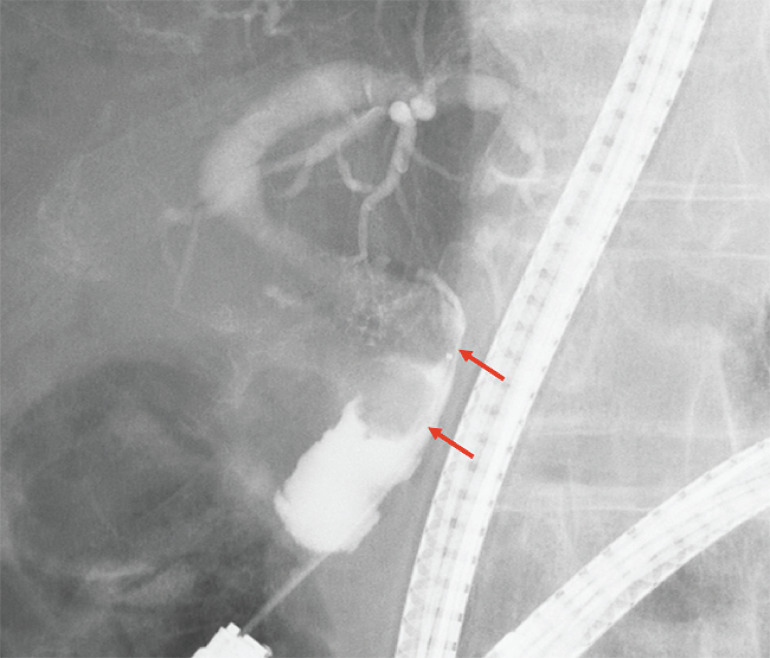
Cholangiographic image showing stones in the common bile duct (red arrows).

**Fig. 5:**
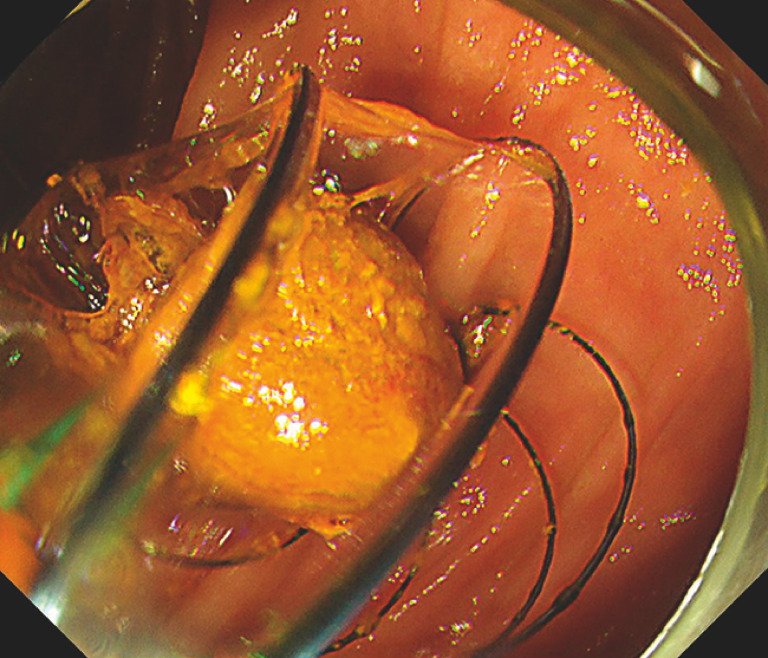
Endoscopic image showing successful stone extraction.

An 81-year-old man who had undergone total gastrectomy with Roux-en-Y for gastric cancer 3 years previously was referred to our facility. Computed tomography and magnetic resonance imaging revealed stones in the common bile duct (CBD) ( Fig. 1 ). Endoscopic retrograde cholangiopancreatography (ERCP) was therefore performed using a short-type single-balloon enteroscope (SIF-H290; Olympus Marketing, Japan) with a working length of 152 cm and a working channel of 3.2 mm in diameter 5 . We also attempted cholangioscopy-guided cannulation using a slim cholangioscope (DRES Slim Scope; Japan Lifeline, Japan) with a length of 195 cm; the diameters of the scope and tip were 2.6 mm and 2.3 mm, respectively ( Fig. 2 ; Video 1 ). Upon reaching the papilla, we employed the slim cholangioscope for cholangioscopy-guided cannulation. When the cholangioscope had been passed through the orifice, we were able to visualize the bile duct mucosa. Subsequently, a 0.025-inch guidewire was advanced, successfully achieving biliary cannulation ( Fig. 3 **a–c** ). POCS ( Fig. 3 **d** ) and cholangiography ( Fig. 4 ) revealed bile duct stones in the CBD. Subsequently, endoscopic sphincterotomy and endoscopic papillary large-balloon dilation were performed, and were followed by successful complete stone extraction ( Fig. 5 ).

Successful cholangioscopy-guided cannulation using a novel slim cholangioscope in a patient who had undergone Roux-en-Y gastrectomy.Video 1

This slim cholangioscope proved effective for cholangioscopy-guided cannulation, despite the patient having undergone Roux-en-Y gastrectomy. Its slender design allowed easy insertion into the scope channel of the balloon enteroscope. This novel slim cholangioscope can aid in the development of effective biliary cannulation techniques in patients with surgically altered anatomy.

Endoscopy_UCTN_Code_TTT_1AR_2AG
